# Improvement of several stress response and sleep quality hormones in men and women after sleeping in a bed that protects against electromagnetic fields

**DOI:** 10.1186/s12940-022-00882-8

**Published:** 2022-07-22

**Authors:** E Díaz-Del Cerro, J Félix, JAF Tresguerres, M De la Fuente

**Affiliations:** 1 Department of Genetics, Physiology and Microbiology (Unity of Animal Physiology). Faculty de Biology, Complutense University of Madrid, José Antonio Novais, 12. 28040, Madrid, Spain; 2 Research Institute of 12 de Octubre Hospital of Madrid (I+12), Madrid, Spain; 3 Department of Physiology. Medicine Faculty, Complutense University of Madrid, Madrid, Spain

**Keywords:** Electromagnetic fields, Hormones, Stress response, Sleep, Men and women, Biological age

## Abstract

**Background:**

The electromagnetic fields (EMFs) emitted by the technologies affect the homeostatic systems (nervous, endocrine, and immune systems) and consequently the health. In a previous work, we observed that men and women, after 2 months of using a bed with a registered HOGO system, that prevents and drain EMFs, improved their immunity, redox and inflammatory states and rejuvenated their rate of aging or biological age. Since, EMFs can act as a chronic stressor stimulus, and affect the sleep quality. The objective of this work was to study in men and women (23–73 years old) the effect of sleeping for 2 months on that bed in the blood concentrations of several hormones related to stress response and sleep quality as well as to corroborate the rejuvenation of their biological age.

**Methods:**

In 18 men and women, plasma concentration of cortisol, dehydroepiandrosterone (DHEA), catecholamines (epinephrine, norepinephrine and dopamine), serotonin, oxytocin and melatonin were analyzed before and after 2 months of using the HOGO beds. A group of 10 people was used as placebo control. In another cohort of 25 men (20 experimental and 5 placebo), the effects of rest on the HOGO system on the concentration of cortisol and testosterone in plasma were studied. In all these volunteers, the biological age was analyzed using the Immunity Clock model.

**Results:**

There is a significant increase in plasma concentration of DHEA, norepinephrine, serotonin, oxytocin, and melatonin as well as in testosterone, after resting for 2 months in that bed with the EMFs avoiding system. In addition, decreases in Cortisol/DHEA and Testosterone/cortisol ratio and plasma dopamine concentration were observed. No differences were found in placebo groups. In all participants that slept on HOGO beds, the biological age was reduced.

**Conclusions:**

Sleeping in a bed that isolates from EMFs and drain them can be a possible strategy to improve the secretion of hormones related to a better response to stress and sleep quality, which means a better endocrine system, and consequently better homeostasis and maintenance of health. This fact was confirmed with the slowdown in the rate of aging checked with a rejuvenation of the biological age.

## Introduction

Electromagnetic radiation has been accompanying living organisms since the dawn of life on Earth. However, their current intensity and omnipresence should be attributed, first of all, to human activity and its technological advances. Due to that fact, humans are constantly exposed to various types of electromagnetic fields (EMFs). On the one hand, all electrically powered devices and transmission lines generate the low frequency (usually 50 or 60 Hz) field. On the other hand, electronic devices, such as mobile phones, television sets or radio transmitters, emit electromagnetic radiation with high frequencies (from 300 MHz to 300 GHz) and these last cause also a thermal effect that can increase the temperature of tissues and organs, causing serious damage to cells [1]. Due to this fact, EMFs exposure has become a major public health issue.

In this context, the last decades, researchers have revealed that electromagnetic fields can produce adverse effects on different regions of human and experimental animal bodies [2, 3]. These effects have been undoubtedly proven in homeostatic systems, the nervous [4–6], endocrine [7, 8] and immune [9] systems, and, consequently, exposure to EMFs can lead to loss of health and increase the risk of suffering pathologies [10]. Moreover, EMFs can affect the sleep regulation [11], being more significant the effect on health of the exposure of EMFs at nighttime than at daytime [12], since nocturnal sleep has a homeostatic role, being necessary for efficient consolidation of cognitive, metabolic and immune functions [13].

The aging process, which starts at the adult age and finishes with the death of the individual, is characterized by a progressive and general deterioration of the functions of the organism, especially those of the homeostatic area such as the nervous, endocrine and immune systems. For this reason, there is a less efficient maintenance of homeostasis and consequently an increase of the risk of illness. Each subject shows a different rate of aging, which constitutes the so called biological age, more relevant to determinate the longevity than chronological age. Biological age is dependent on genes, but in a higher proportion on the environment and life style [14]. The hormonal response to stress is one of the factors involved in this rate of aging [15, 16]. In fact, the adequate hormonal adaptation to stress situations is one of the hallmarks of health [17]. In this context, the EMFs are a relevant stress factor [3, 18–20] and, consequently, through autonomic and neuroendocrine responses, are able to affect the health state [21].

Following this line, stress is one of the main factors influencing sleep [22, 23], so high levels of stress lead to a poor sleep. Besides, during aging, both an inadequate stress response [14] and a poor sleep are highly prevalent [24], affecting health and life expectancy [25–28]. Sleep quality is also affected by EMFs emitted from human modern technologies [29, 30] and EMF exposure at night has more severe effects on health in comparison to EMF radiation during the daytime [12].

Regarding the neuroendocrine system, this is markedly affected by inadequate sleep [24], having both, neuroendocrine system and sleep, close bidirectional connections [31, 32]. Many neurotransmitters and hormones are involved in sleep regulation [33], being the most studied those involved in the stress response [34], such as cortisol, dehydroepiandrosterone (DHEA) and catecholamines. But other hormones are also closely linked to sleep, such as serotonin [35], oxytocin [36], testosterone [37] and, especially, melatonin [38].

In a previous paper, we observed that both men and women,, improved their immunity, redox and inflammatory states and rejuvenated there biological age after 2 months of using a bed with a registered HOGO system that protects for EMFs [39] and additionally drains it from the body. Therefore, it was shown that the immune system, one of the homeostatic systems of the organism was markedly improved. Thus, we set out to investigate the effect of this strategy on another of the homeostatic areas, the endocrine system. In order to proceed with the above mentioned idea, the aim of the present study was to investigate the effect of sleeping in a bed with a registered HOGO system that avoids from EMFs, and also drain them, on the concentration of various hormones related to stress and sleep quality,

## Material and methods

### Study groups and experimental design

The participants were 28 men and women (23-73 years old), divided into two groups: an experimental group using HOGO system beds, which isolate from EMFs and drain the existing ones (*n*=18), and a placebo group (*n*=10), which slept on normal beds. In addition, we studied the effect of sleeping on the HOGO rest system on the concentration of cortisol and testosterone in plasma in another cohort of men (*N*=25) aged 23-73 years, which were divides into the following experimental groups: Experimental group (*n*=20) and Placebo group (*n*=5). All of the participants had similar characteristics regarding social class (upper middle), education (the majority with Bachelor´s degree) and lifestyle (sport, eating habits and smoking).

**Fig. 1:**
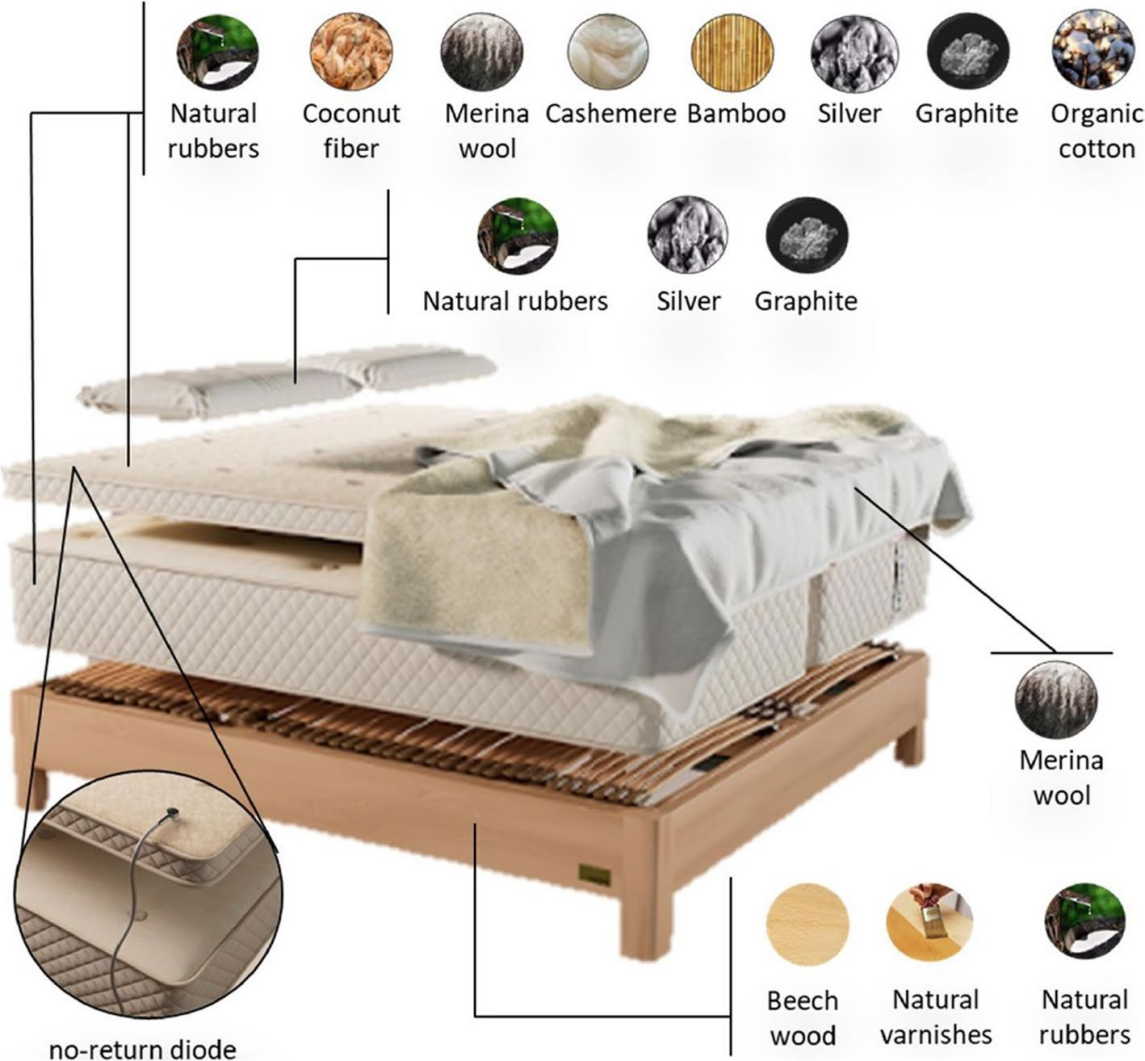
HOGO system structure and materials

HOGO beds (HOGO Company) consist of an articulated box spring, customized, with pivoting and adaptation system for each part of the body, laminated and vaporized beech wood, natural varnishes and natural rubbers as part of the laminate support. The mattress and topper are composed of natural rubbers, coconut fiber, Merina wool, cashemere, bamboo, silver, graphite, organic cotton, and patented technology for the removal of EMFs. The pillow is composed of natural latex, woven with graphite and silver mesh and patented technology for the removal of EMFs. Finally, the blanket is made of Merina wool tested and flaky to prevent allergies and improve perspiration and oxygenation. The HOGO rest system is connected to the building grounding system for the removal of residual and accumulated EMFs in the person during the day. The plug, with a no-return diode, is made of plastic and rubber so as not to be a conductor of the electricity. In addition, normal beds are visually equal but without patented technology for the removal of EMFs and made with not-100%-naturals materials and with metallic springs (Fig. 1).

The volunteers were randomly assigned to a standard bed or the HOGO rest system. In none of the cases the volunteers were compensated with money, and they received the standard bed or the HOGO system to be used at home. Both standard and HOGO beds were installed in the room of each volunteer, without making any other changes in this room in order to allow them to continue with their usual lifestyle. The electromagnetic fields were measured throughout the room (walls, furniture, floor, windows...) before and after plugging in the system in the sleeping area. Thus, Electromagnetic fields in low frequencies were measured using ME3830B analyzer (Gigahertz solutions) with a frequency range of 16 Hz-100 KHz, magnetic flux density (one-dimensional): 1-1999 nT and electric field strength: 1-1999 V/m. High-frequency electromagnetic radiation was measured by HF35C analyzer (Gigahertz solutions) with a frequency range of 800 MHz - 2.7 GHz and a power flux density of 0.1-1999 μW/m^2^. The EMF measurements in the room were 990±270 mV before plugging HOGO system, and 35±10 mV, after, decreasing until reaching no concern values according to Building Biology Evaluation Guidelines for Sleeping Areas SBM-2008 [40].

Before and after resting for two months on these beds, 12 mL of blood were obtained from each participant between 9:00-10:00 a.m. The study design, which was approved by the Human Ethics Committee of the 12 de Octubre Hospital of Madrid, was explained to the participants, and written consent was obtained from each. Exclusion criteria were: 1) Severe and unstable medical conditions, or a history of chronic diseases; 2) aphasia, confusion, or psychiatric comorbidity; 3) taking medications, such as anti-inflammatory agents, muscle relaxants, corticoids and antidepressants; 4) any previously diagnosed sleep diseases, including narcolepsy, periodic limb movement disorder, obstructive sleep apnea; 5) previous surgery; 6) pregnancy; 7) electrohypersensitivity; 8) and non-cooperation during the evaluation.

### Collection of human plasma samples

Peripheral blood samples (12 mL) were collected using vein puncture and sodium citrate-buffered Vacutainer tubes (BD Diagnostic, Spain), between 8:00 to 9:00 a.m. to avoid circadian variations. Blood samples were centrifuged at 1300 g for approximately 20 minutes. Then, plasma was separated from total blood cells and frozen at -80ºC until use.

### Hormonal assays

Plasma cortisol was measured by using a Cortisol ELISA kit (ADI-900-071, Enzo Life Sciences, UK), plasma DHEA was determined by a DHEA ELISA kit (ADI-900-093, Enzo Life Sciences, UK), plasma catecholamines (epinephrine: E, norepinephrine: NE, dopamine: DP) was quantified by 3-CAT ELISA kit (BA E-6600, LDN Labor Diagnostika, Germany). Plasma oxytocin and serotonin were determined by a Oxytocin ELISA kit (ADI-900-153A-0001, Enzo Life Sciences, UK) and Serotonin ELISA kit (ADI-900-175, Enzo Life Sciences, UK), respectively. Also, plasmatic concentration of testosterone and melatonin were measured using a Free Testosterone ELISA Kit (CAN-FTE-260, Diagnostics Biochem Canada Inc) and a Melatonin ELISA kit (E4630-100, BioVision), respectively.

The results were expressed in µg cortisol/mL of plasma, µg DHEA/mL of plasma, pg E/mL of plasma, pg NE/mL of plasma, pg DP/mL of plasma, ng Oxytocin/mL of plasma, ng serotonin/mL of plasma, pg testosterone/mL of plasma and pg melatonin/mL of plasma.

### Immunity clock model: biological age analysis

Biological age or ImmunolAge was calculated using a mathematical model generated with the determination of the following immune functions: chemotaxis of neutrophils and lymphocytes, neutrophil phagocytosis, lymphocyte proliferation and *Natural Killer* (NK) cytotoxic activity [41]. Thus, Immunity Clock formula for the estimation of the ImmunolAge is:

$${\varvec{I}}{\varvec{m}}{\varvec{m}}{\varvec{u}}{\varvec{n}}{\varvec{o}}{\varvec{l}}{\varvec{A}}{\varvec{g}}{\varvec{e}}=93.943-0.230\times Natural killer activity$$

$$-0.001\times PHA-stimulated lymphoproliferation$$

$$-0.022\times Neutrophil chemotaxis$$

$$-0.02\times Neutrophil phagocytic index$$

$$-0.019\times Lymphocyte chemotaxis$$

#### Neutrophil and lymphocyte isolation

Neutrophils and lymphocytes were isolated by a Ficoll Histopaque density gradient of 1.119 and 1.077 densities (Sigma-Aldrich, Spain). The cells collected (determining viability by blue tripan staining) are adjusted to the corresponding final concentration for the development of each test.

#### Chemotaxis capacity

The chemotaxis capacity of the cells was realized by a modification of the Boyden method (1962) [42] previously described [43]. Boyden chambers have two compartments separated by a filter in which the cells that move to the lower chamber towards the chemoattractant (formyl peptide (fMLP)) are counted. The Neutrophil and Lymphocyte Chemotaxis Index was evaluated.

#### Phagocytic capacity

The phagocytic capacity of neutrophils was performed following the method based on the ability of phagocytic cells to ingest inert compounds (latex balls) [43]. The Phagocytic Index (number of latex particles ingested per 100 neutrophils) was evaluated.

#### *Natural Killer* activity

It was evaluated according to the method based on the measurement by colorimetry of the lysis of the target cells (human tumor line) by determining the enzyme lactate dehydrogenase (LDH), using tetrazolium salts (Cytotox 96 TM kit, Promega) [43]. The results were expressed in percentage of lysis of the target cells.

#### Lymphoproliferative capacity

The proliferation capacity of lymphocytes was evaluated in cultures carried out both at basal and stimulated with phytohemagglutinin (PHA) conditions [43]. The results were calculated as the amount of 3H-thymiine (counts per minute, c.p.m.) for basal and stimulated conditions, and were expressed in lymphoproliferation capacity (%) giving the value 100 to the c.p.m. obtained at baseline.

### Statistical analysis

Statistical analysis was performed in SPSS IBM, version 21.0 (SPSS, Chicago, IL, USA). Data are presented as mean ± standard deviation (SD). Normality of the samples and homogeneity of the variances were checked using the Kolmogorov–Smirnov test and Levene test, respectively. Comparisons between the groups were made by the independent-samples t-test according to the compatibility of the data with normal distribution and comparisons between results of a same group were made by the dependent-samples t-test and p ≤ 0.05 was considered statistically significant. Figures were built using GraphPad Prism 8 Software (LLC, San Diego, CA, USA).

## Results

In order to evaluate the effects of using a bed with a EMFs-avoiding system together with EMFs-drain on the hormonal state, several neurotransmitters and hormones were measured in the plasma of the volunteers.

**Fig. 2:**
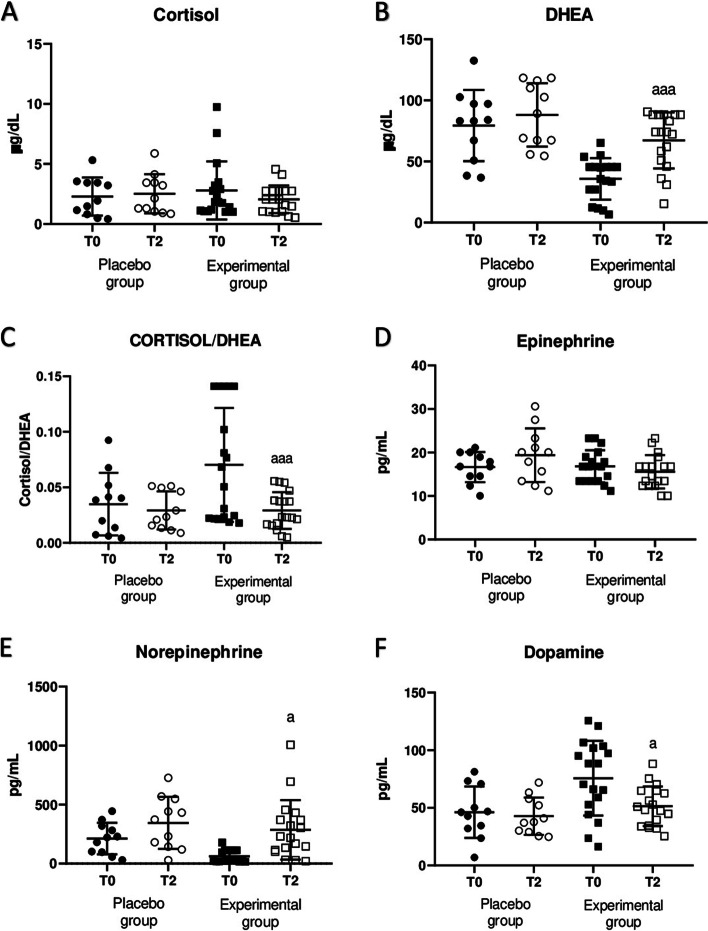
Concentration of stress response hormones in plasma samples before (T0) and after sleeping for 2 months (T2) using the HOGO system to avoid EMFs (Experimental group, *n* = 18) or normal beds (Placebo group, *n* = 10). **A** Concentration of cortisol in µg/dL; **B** Concentration of DHEA in µg/dL; **C** Cortisol/DHEA ratio; **D**) Concentration of epinephrine in pg/mL; E) Concentration of norepinephrine in pg/mL; F) Concentration of dopamine in pg/mL. a: *p* < 0.05, aaa: *p* < 0.001 respect to the values obtained at T0

**Fig. 3:**
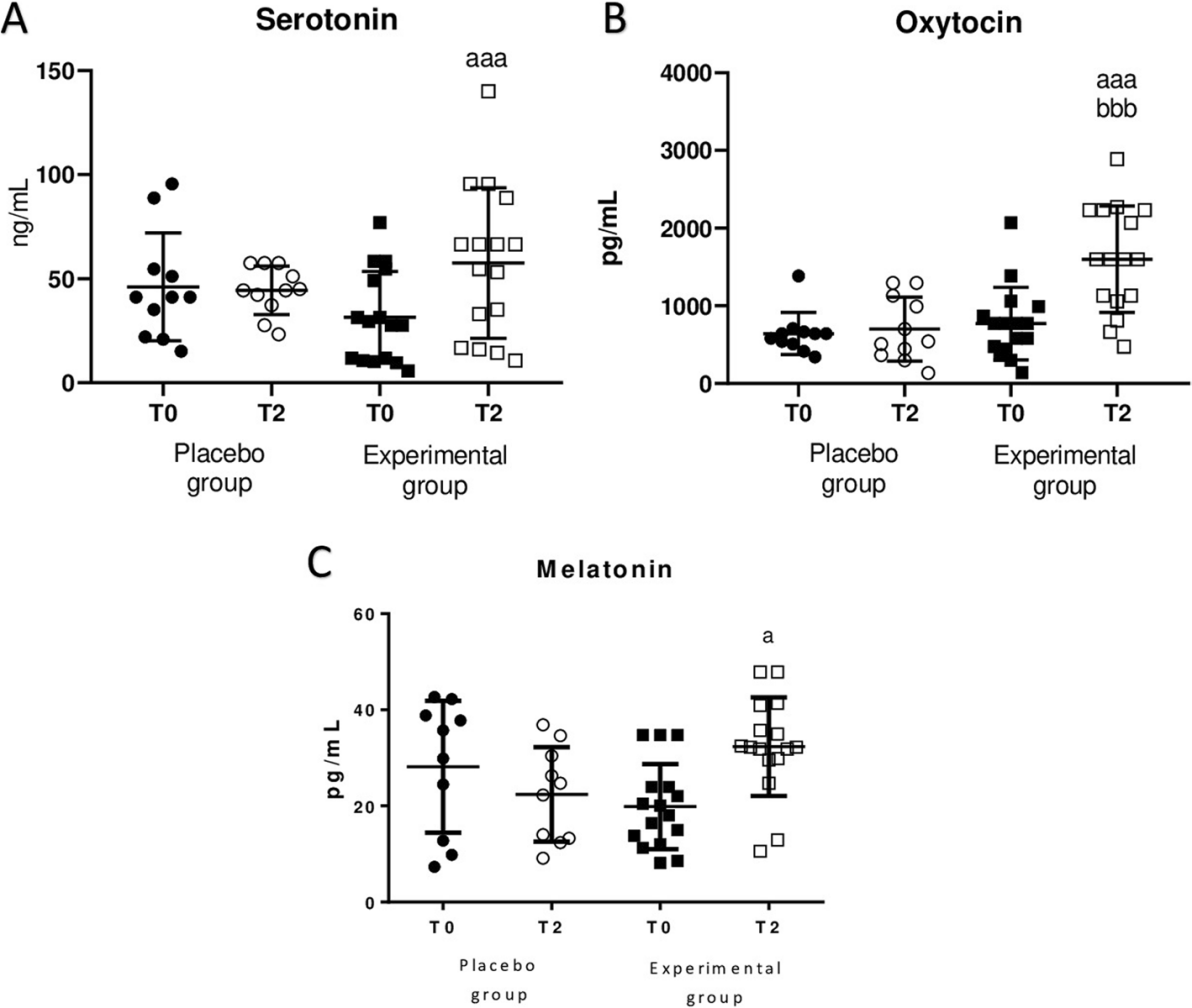
Concentration of serotonin, oxytocin and melatonin in plasma samples before (T0) and after sleeping for 2 months (T2) using the HOGO system to avoid EMFs (Experimental group, *n* = 18) or normal beds (Placebo group, *n* = 10). **A** Concentration of serotonin in ng/mL; **B** Concentration of oxytocin in pg/mL; **C** Concentration of melatonin in pg/mL. a: *p* < 0.05; aaa: *p* < 0.001 respect to the values obtained at T0

In the following results (Fig. 2, Fig. 3 and Fig. 5A), men and women data from each experimental group were not separated since no statistically significant differences were found due to sex in any of the parameters investigated.

The results obtained for stress hormones concentration, cortisol, DHEA and catecholamines, in plasma as well as their ratio, are shown in Fig. 2. As can be seen, after two months of resting in the HOGO system, subjects in the experimental group showed a very significant increase in plasma concentrations of DHEA (*p* < 0,01; Fig. 2B). Although there was a decrease in the values of cortisol concentration, this reduction was not statistically significant (Fig. 2A). Nevertheless, the cortisol/DHEA ratio decreased significantly after bed use time with the EMFs protection/ drain system (*p* < *0.05;* Fig. 2C), compared to the values obtained at T0. However, no differences in the concentrations of any of these hormones were observed in the Placebo group between BASAL intake and the two months of standard bed use.

In addition, the results obtained for catecholamines, plasma concentration of norepinephrine increased significantly (Fig. 2E, *p* < 0.05) after sleeping for two months on HOGO bed, whereas dopamine concentration decreased (Fig. 2F, *p* < 0.05). However, no changes were found in epinephrine concentration in plasma. In the placebo group no differences were observed.

Furthermore, the results obtained for serotonin, oxytocin and melatonin concentration in plasma are shown in Fig. 3. Hormones, serotonin (Fig. 3A, *p* < 0.001), oxytocin (Fig. 3B, *p* < 0.001) and melatonin (Fig. 3C, *p* < 0.05), increased significantly after sleeping for two months on HOGO bed. In the placebo group no differences were observed.

**Fig. 4:**
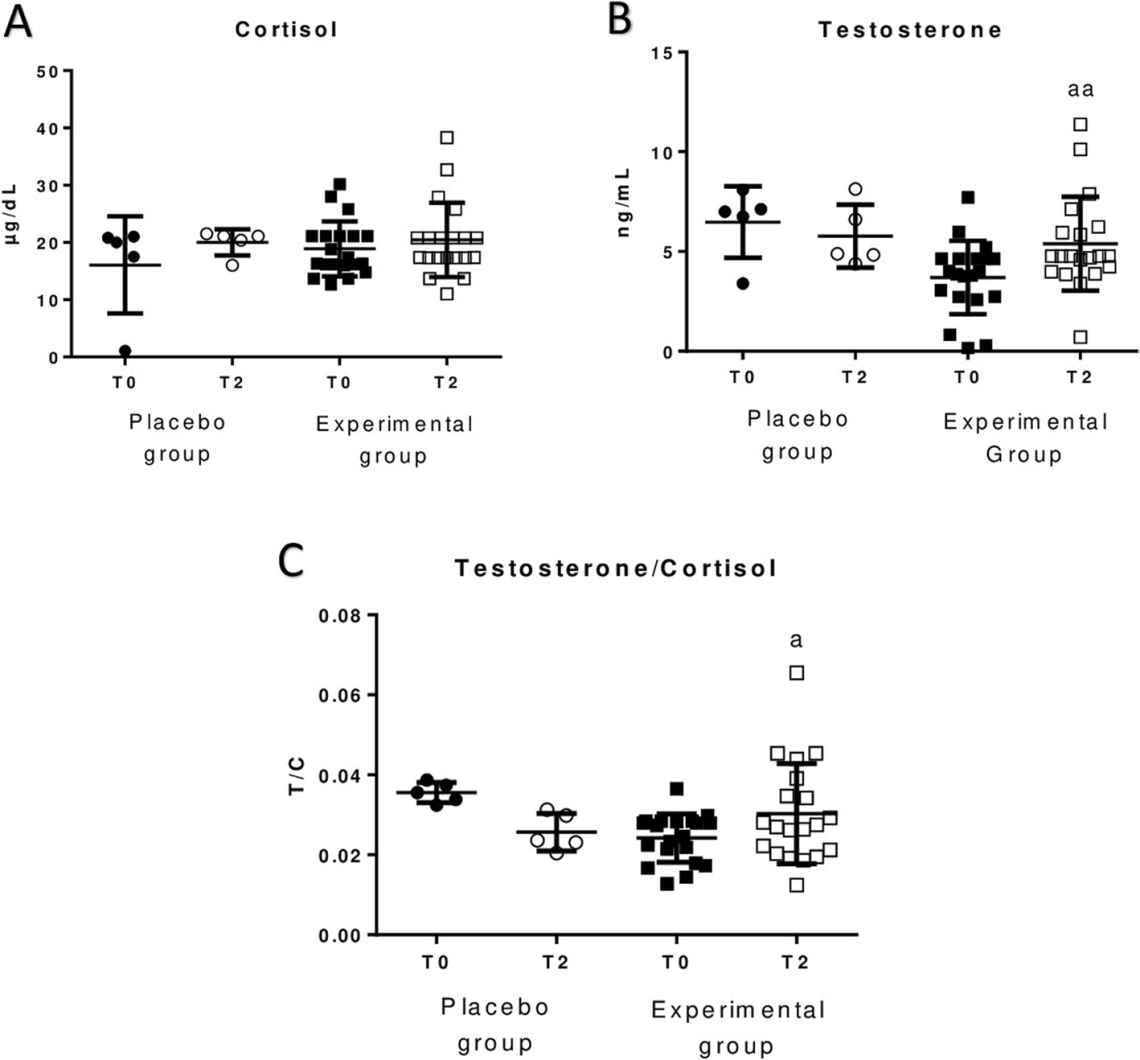
Concentration of cortisol, testosterone and testosterone/cortisol ratio in plasma samples of men before (T0) and after sleeping for 2 months (T2) using the HOGO system to avoid EMFs (Experimental group, *n* = 20) or normal beds (Placebo group, *n* = 5). **A** Concentration of cortisol in µg/dL; **B** Concentration of testosterone in ng/mL; **C** Testosterone/cortisol (T/C) ratio. a: *p* < 0.05; aa: *p* < 0.01; aaa: *p* < 0.001 respect to the values obtained at T0

The Fig. 4 shows the results obtained for cortisol (Fig. 4A), testosterone (Fig. 4B) and testosterone/cortisol ratio (Fig. 4C) in plasma of men after sleeping on beds with HOGO technology. Plasma testosterone concentration (*p* < 0.01) and testosterone/cortisol ratio (*p* < 0.05) increased significantly after sleeping for two months on the HOGO bed. However, no differences were found in cortisol concentration in plasma. In placebo group no differences were observed.

**Fig. 5:**
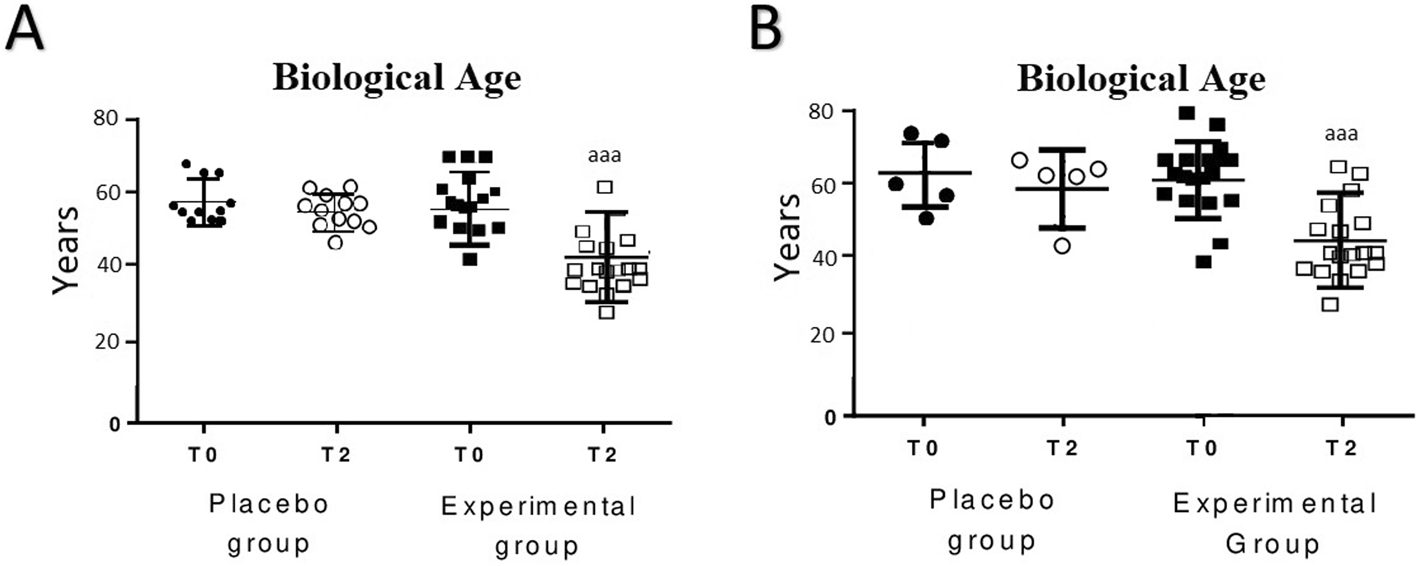
Biological age before (T0) and after sleeping for 2 months (T2) using the HOGO system to avoid EMFs. **A** In the cohort of men and women (Experimental group, *n* = 18; Placebo group, *n* = 10); **B** In the cohort of men (Experimental group, *n* = 20; Placebo group, *n* = 5). aaa: *p* < 0.001 respect to the values obtained at T0;

The results of the biological age are shown in Fig. 5, observing that after sleeping for 2 months on the HOGO system bed, biological age decreased significantly (*p* < 0.001) in both cohorts (Fig. 5A: cohort of both men and women; Fig. 5B: cohort of men). No differences were observed in the placebo groups.

## Discussion

In the present study, for the first time, it is shown that men and women sleeping for two months in a bed made with natural materials and with an electromagnetic field isolation and drain system, have improved several neurotransmitters and hormones related to stress response and to the quality of sleep. This indicates that such a rest system has been able to improve people's health. In fact, in order to maintain health, it is necessary to have adequate homeostatic regulators, such as the nervous, endocrine and immune systems, as well as their intercommunication [44]. In a previous study [39] it was already shown that the use of this bed for two months was able to improve immune function, as well as the oxidative and inflammatory state, all this followed by a rejuvenation of the biological age, that is, of the rate of aging of the individuals [43, 45].

The EMFs produced by modern technologies affect quality of sleep, being these EMFs a stress factor [3, 18–20]. Both sleep and stress are situations in which the three homeostatic systems work together [46], and they can be affected by EMFs [47–50]. Thus, the use of a bed with EMFs-isolation-and-drain system, which improves immune system function [39] can also do that with the neuroendocrine system. In fact, this has been observed in the present work.

Sleeping in the HOGO system increased plasma DHEA concentration, a hormone that antagonizes the effects of glucocorticoids [51]. Having higher DHEA concentration means, not only being better prepared against stress, but also working as neuroprotective and preventing a long list of disorders and pathologies, given the beneficial characteristics of this hormone [52]. The relationship of plasma concentrations of cortisol and DHEA has been proposed when elevated, as a marker of morbidity and mortality [53]. In fact, lifestyle strategies that can reduce the first and increase the second hormone, such as physical exercise [54, 55] or dietary supplementation [56, 57], have been proposed as suitable for a better health maintenance. In the present study, sleeping for two months in the HOGO bed decreases significantly the cortisol/DHEA ratio thus playing in the right direction.

Other stress hormones studied in the present work were the catecholamines, such as epinephrine, norepinephrine and dopamine. Norepinephrine is closely associated with the quality of sleep and its maintenance. Although an increase of the activation of the sympathetic nervous system and, consequently, the production of greater amounts of norepinephrine, may seem like an unfavorable fact for health, von Känel et al. (2018) [58] found that people with sleep disorders had significantly lower plasma norepinephrine concentrations. This fact seems to be associated with an increase in fibrinogen and other inflammatory molecules. In the present study, participants who rested on the HOGO beds experienced an increase in plasma concentrations of this hormone, which could be related to lower degree of inflammation.

In fact, we have found that people who slept for two months in that bed experience a decrease in their pro-inflammatory cytokines [39]. Exposure to EMFs can also affect catecholamine secretion [19], and specifically cause a decrease in norepinephrine concentration [59].

Dopamine is a neurohormone with many beneficial properties [60]. However, research on the effects of sleep deprivation on this hormone has shown that these sleep disturbances are associated with an increase in plasma dopamine concentration [61]. However, dopamine levels decrease after sleeping in the HOGO rest system. Thus, the property of the bed to isolate or to drain these EMFs during the night may be one of the ways that has allowed obtaining the present results.

With respect to serotonin and oxytocin, in the present paper plasma concentrations of these hormones were increased after sleeping in HOGO beds. The positive effects of serotonin on many functions of the body are widely known [62]. Thus, the increase in this hormone after two months of using the HOGO bed will mean better mood, stress control and a lower risk of depression and other psychiatric problems [63]. Also, given the transformation of serotonin into melatonin and its relation with a better circadian rhythm and sleep quality [64], this transformation could facilitate these beneficial effects.

The increases of oxytocin, given its role in the regulation of nociception, its pro-social, anxiolytic, and anti-stress behavioral and physiological effects [65, 66], represents significant improvements in these aspects. In addition, the antioxidant and anti-inflammatory effects of this hormone [67] allow us to understand that not only that increase mean better health, it also promotes a slower rate of aging, avoiding the pathological risks associated with it.

In the sleep cycle, melatonin is one of the major regulators. In general, plasma melatonin levels are lower during the day and higher at night [68]. In the present study, melatonin levels in plasma, in the morning, increase after sleeping on HOGO beds. This can be the reason, at least in part, for the improvement in immune functions and inflammatory state observed in volunteers who slept for 2 months on HOGO beds in a previous study carried out by our laboratory [39], since melatonin has been shown antioxidant, immunomodulatory, and anti-inflammatory effects [69]. Moreover, it has been proposed that melatonin acts, together with other hormones such as norepinephrine, cortisol and cytokines such as IL-1 beta, in a network influencing stress responses [46]. We measured melatonin levels between 8–9 a.m., and although the pick of this hormone is between 2–4 a.m. and the minimum levels were reached during the afternoon, in the studied period melatonin levels were still relatively high, as compared with the controls [70].

As mentioned above, one of the objectives of this study was to investigate the effect of resting in a bed with technology capable of isolating/draining from EMFs on some hormones related to the stress response. For this reason, we wanted also to study the concentration of testosterone in plasma, as well as its relationship with cortisol, in a cohort of men since it has been shown to act in them as markers of stress [71]. In this study, it can be observed that the concentration of testosterone in plasma and the testosterone/cortisol ratio increased after two months of using the HOGO beds. Several studies have reported that testosterone in plasma decreases after the exposure to EMFs, in mouse [72] and human [73], as a consequence of poor sleep [74]. Since continued exposure to EMFs leads to deterioration in sleep quality and quantity [75], the results obtained in the present study seem to indicate that sleep on HOGO system has been able to improve testosterone concentrations due to its EMFs-avoiding/draining effect and, consequently, by establishing a better sleep process. In fact, reproductive organs, especially in the male, are very sensitive to environmental stresses such as EMFs due to radiofrequency electromagnetic radiation [76]. Moreover, these results are linked to the increase observed also in melatonin in the present study since the antioxidant effect of melatonin seems to help to prevent that damage in reproductive organs [77], although the results were not obtained in the same people.

In this type of strategy in humans the existence of a placebo effect is possible [78]. This possibility was analyzed in the present study, incorporating a group that slept for two months in a similar bed but without the isolating properties from EMFs. Curiously, none of the hormones showed any change in this group, which shows that the observed effects are not a consequence of people's belief in the benefit they will obtain from using the bed, but are due to the special characteristics of the HOGO system.

In addition, in a previous study it was observed that people who slept for two months in the HOGO system had their biological age rejuvenated, that is, their natural aging process was slowed down [39]. Thus, this improvement in testosterone concentration and the testosterone/cortisol ratio in the present paper may reflect that rejuvenation observed since with aging the concentration of testosterone in plasma decreases, whereas the testosterone/cortisol ratio increases,. Therefore, EMFs exposure is associated with an accelerated aging, leading to a shorter longevity from 80 years to 65–70 [79]. In this line, in a previous study, we have demonstrated that sleeping on HOGO beds was able to rejuvenate the biological age [39]. This effect is corroborated with the results obtained in the present study, reinforcing the idea that sleeping isolated from EMFs slows down the rate of aging.

Our study has several limitations. One of these limitations is the sample size used. A larger number of participants should have allowed studying the differences due to characteristics of profession, social class or environmental factors (such as room light, noise…), but this was not possible since the manufacturing of HOGO beds is very expensive. Another limitation is that the long-term effect of resting on HOGO beds was not investigated and could be interesting to check whether after a year, for example, the effects on these hormones are maintained. Finally, the measurement of melatonin in the morning represents another limitation of the study since this hormone reaches its maximum peak during the night.

With these limitations in mind, the present study to corroborate the previous results on the positive effects of this EMF-avoiding/ draining strategy to rejuvenate the biological age and it provides evidences for its role improving the secretion of a series of hormonal compounds related to a better response to stress and sleep quality, which involve better maintenance of homeostatic systems and, consequently, health and general well-being.

## Data Availability

All data generated or analyzed during this study are included in this published article.

## Abbreviations

EMFs: Electromagnetic fields
Hz: Hertz
KHz: Kilohertz
MHz: Megahertz
GHz: Gigahertz
nT: Nanotestla
mV: Milivolt
μW/m^2^: Microwatt/square meter
DHEA: Dehydroepiandrosterone
E: Epinephrine
NE: Norepinephrine
DP: Dopamine
pg/mL: Picogram/milliliter
µg/mL: Microgram/milliliter
T0: Initial time
T2: After sleeping on normal beds or on HOGO beds
NK: *Natural killer*
fMLP: Formylated peptid
LDH: Lactate dehydrogenase
PHA: Phytohaemagglutinin
c.p.m: Counts per minute
T/C: Testosterone/cortisol ratio
